# Development and validation of an inflammatory-immune-nutritional integrated scoring system: a novel strategy for predicting postoperative survival in non-small cell lung cancer

**DOI:** 10.7717/peerj.21122

**Published:** 2026-04-06

**Authors:** Qing Huang, Nie Xu, Ke Xu

**Affiliations:** 1 Department of Radiotherapy, Sichuan Cancer Hospital & Institute, Sichuan Cancer Center, School of Medicine, University of Electronic Science and Technology of China, Sichuan, China; 2 Department of Oncology, Chengdu First Peoples’ Hospital, Integrated TCM & Western Medicine Hospital, Chengdu University of Traditional Chinese Medicine, Sichuan, China

**Keywords:** Nutritional, Immune, Inflammatory, Non-small cell lung cancer

## Abstract

**Background:**

Accurate prognosis prediction is crucial for managing non-small cell lung cancer (NSCLC). Tumor heterogeneity limits the predictive power of traditional tumor lymph node metastasis (TNM) staging alone. The interplay between inflammation, immunity, and nutrition in the tumor microenvironment offers a potential source of novel prognostic biomarkers.

**Methods:**

This multicenter retrospective study enrolled 473 stage I-IIIA NSCLC patients who underwent radical resection. An inflammatory-immune-nutritional integrated scoring system (IINS) was developed using Least Absolute Shrinkage and Selection Operator (LASSO) Cox regression from preoperative hematological parameters. The model was validated internally (*n* = 142) and externally using the NHANES database (*n* = 134). A prognostic nomogram incorporating IINS and clinicopathological factors was constructed and evaluated using time-dependent receiver operating characteristic (ROC) curves, calibration plots, and decision curve analysis (DCA).

**Results:**

The IINS was formulated based on platelet-to-lymphocyte ratio (PLR), systemic inflammatory response index (SIRI), and prognostic nutritional index (PNI). Patients were stratified into high- and low-IINS groups using an optimal cutoff of 4.9. Multivariate analysis identified IINS as an independent prognostic factor for both overall survival (OS) (HR = 1.63, 95% CI [1.17–2.26], *P* = 0.004) and disease-free survival (DFS) (HR = 1.41, 95% CI [1.02–1.97], *P* = 0.039). The nomogram demonstrated good predictive accuracy, with 1-, 3-, and 5-year area under the curves (AUCs) for OS ranging from 0.695 to 0.730 in the validation cohort. DCA confirmed the nomogram’s superior clinical utility compared to the TNM staging system.

**Conclusions:**

The novel IINS, integrating inflammatory, immune, and nutritional status, is a robust independent prognostic indicator for resected NSCLC. The developed nomogram provides a user-friendly and accurate tool for predicting postoperative survival, potentially aiding in risk stratification and personalized adjuvant therapy decisions.

## Introduction

Lung cancer remains a critical global health challenge, exhibiting the highest incidence and mortality rates among all malignancies (Sung et al., 2021). Non-small cell lung cancer (NSCLC) relies heavily on precise prognostic frameworks to guide clinical outcomes and therapeutic strategies (Ettinger et al., 2021). Current standardized management protocols for early-stage NSCLC (stage I-IIIA) prioritize radical surgical resection as the cornerstone of treatment. Supported by the widespread adoption of lung cancer screening technologies, advancements in surgical techniques, and standardized adjuvant therapeutic regimens, these approaches have significantly improved 5-year survival rate (Treasure, 2019). However, clinical data indicate that over 20% of postoperative patients experience locoregional recurrence or distant metastasis despite strict adherence to established clinical guidelines, representing a critical barrier to long-term survival (Zhang et al., 2018; Fick et al., 2025). Current prognostic systems for NSCLC predominantly rely on conventional parameters, including tumor lymph node metastasis (TNM) staging, surgical margin status, lymphovascular invasion, histologic classification, and driver gene mutation profiles (Rami-Porta et al., 2024; Kris et al., 2017; Romero-Ventosa et al., 2015). Notably, tumor heterogeneity-manifested across genomic, proteomic, and phenotypic levels directly drives substantial variability in therapeutic responses and prognostic outcomes among patients with identical clinicopathological characteristics (McGranahan & Swanton, 2017; Dagogo-Jack & Shaw, 2018). The intricate relationship between these biological characteristics and clinical outcomes underscores the necessity of integrating multidimensional clinical data to identify biomarkers with predictive value. Such biomarkers could significantly enhance existing evaluation frameworks, thereby facilitating the realization of personalized and precise therapeutic strategies.

The interplay mechanisms of inflammation, immunity, and nutrition within the tumor microenvironment have emerged as a critical focus in prognostic research. Evidence demonstrates that systemic inflammatory responses and immunosuppressive states drive tumor progression by promoting angiogenesis, epithelial-mesenchymal transition, and other pathways (De Martino et al., 2024; De Visser & Joyce, 2023), while the resultant immunosuppressive microenvironment critically regulates malignant behaviors such as tumor proliferation, invasion, and metastasis (Mantovani et al., 2008; Grivennikov, Greten & Karin, 2010). Clinical studies have identified various inflammatory markers and immune parameters in peripheral blood as crucial biomarkers for prognostic evaluation in NSCLC. These include white blood cell count (WBC) (Jeong et al., 2017), neutrophil count (NEUT) (Teramukai et al., 2009), platelet count (PLT) (Sandfeld-Paulsen, Aggerholm-Pedersen & Winther-Larsen, 2023), C-reactive protein (CRP) (Hara et al., 2007), neutrophil-to-lymphocyte ratio (NLR) (Jiang et al., 2019), platelet-to-lymphocyte ratio (PLR) (Zhao et al., 2016), lymphocyte-to-monocyte ratio (LMR) (Wang et al., 2019; Zhai et al., 2021), systemic immune-inflammation index (SII) (Nøst et al., 2021), advanced lung cancer inflammation index (ALI) (Taylor et al., 2024), systemic inflammatory response index (SIRI) (Hu et al., 2020), and lymphocyte count (LY) (Eberst et al., 2022).

Within the tumor biological context, metabolic dysregulation-induced nutritional imbalance represents a critical area of investigation. Patients with malignancies frequently manifest malnutrition-related phenotypes, including hypoalbuminemia and reduced body mass index (BMI), attributable to tumor-associated metabolic reprogramming and treatment-related toxicity (Nitenberg & Raynard, 2000). Notably, nutritional status serves not only as a pivotal biomarker for therapeutic tolerance but also as an independent prognostic predictor distinct from conventional clinical parameters (Martin et al., 2015; McMillan, 2013). The prognostic relevance of preoperative nutritional assessment tools to postoperative complications and survival outcomes has been validated across multiple cancer types (Hayasaka et al., 2020; Takamori et al., 2017; Hino et al., 2020; Li et al., 2023). Malnutrition induces immunosuppression and amplifies inflammatory cascades, while inflammation reciprocally depletes nutritional reserves (*e.g.*, albumin) and impairs immune competence, collectively driving tumor progression and metastatic dissemination. Inflammation, immunity, and nutrition constitute a multileveled dynamic regulatory network within the malignant tumor microenvironment, interacting through bidirectional feedback mechanisms to collectively govern tumorigenesis, progression, therapeutic response, and clinical outcomes. Building on these pathophysiological insights, this study developed a multidimensional Inflammatory-Immune-Nutritional Scoring System (IINS) by integrating systemic inflammatory markers, immune profiling parameters and nutritional assessment indices. Synergistically incorporating clinicopathological characteristics, this framework establishes a personalized prognostic nomogram model, offering a visual stratification tool for recurrence evaluation framework to support evidence-based postoperative treatment decisions in NSCLC.

## Materials & Methods

### Patients

This investigation was designed as a multicenter retrospective cohort study and included individuals with pathologically confirmed stage I-IIIA NSCLC who underwent curative-intent surgery at Sichuan Cancer Hospital (*n* = 518) and Chengdu First People’s Hospital (*n* = 214) between January 2018 and January 2021. Eligible patients were required to have undergone complete (R0) resection and to have a definitive postoperative histopathological diagnosis of NSCLC. Additional inclusion criteria were the absence of any neoadjuvant treatment prior to surgery, including chemotherapy, radiotherapy, targeted therapy, or immunotherapy, and the availability of a complete set of preoperative laboratory results encompassing hematology, liver and renal function tests, and metabolic profiles. Patients who had received blood transfusions or nutritional supplementation within three months prior to surgery were excluded to avoid confounding systemic parameters.

Further exclusion criteria comprised a documented history of hematologic malignancy or immune system disorders, active infections or systemic inflammatory conditions at the time of surgery, synchronous primary malignancies, incomplete clinical or follow-up data, and loss to follow-up during the observation period. After applying these criteria, a total of 473 patients (305 from Sichuan Cancer Hospital and 168 from Chengdu First People’s Hospital) were included in the final analysis, and all tumors were staged according to the eighth edition of the American Joint Committee on Cancer (AJCC) staging system (Goldstraw et al., 2016). The entire cohort was randomly divided into a training set and an internal validation set using a 7:3 allocation ratio to enable model construction and testing under comparable conditions.

**Figure 1:**
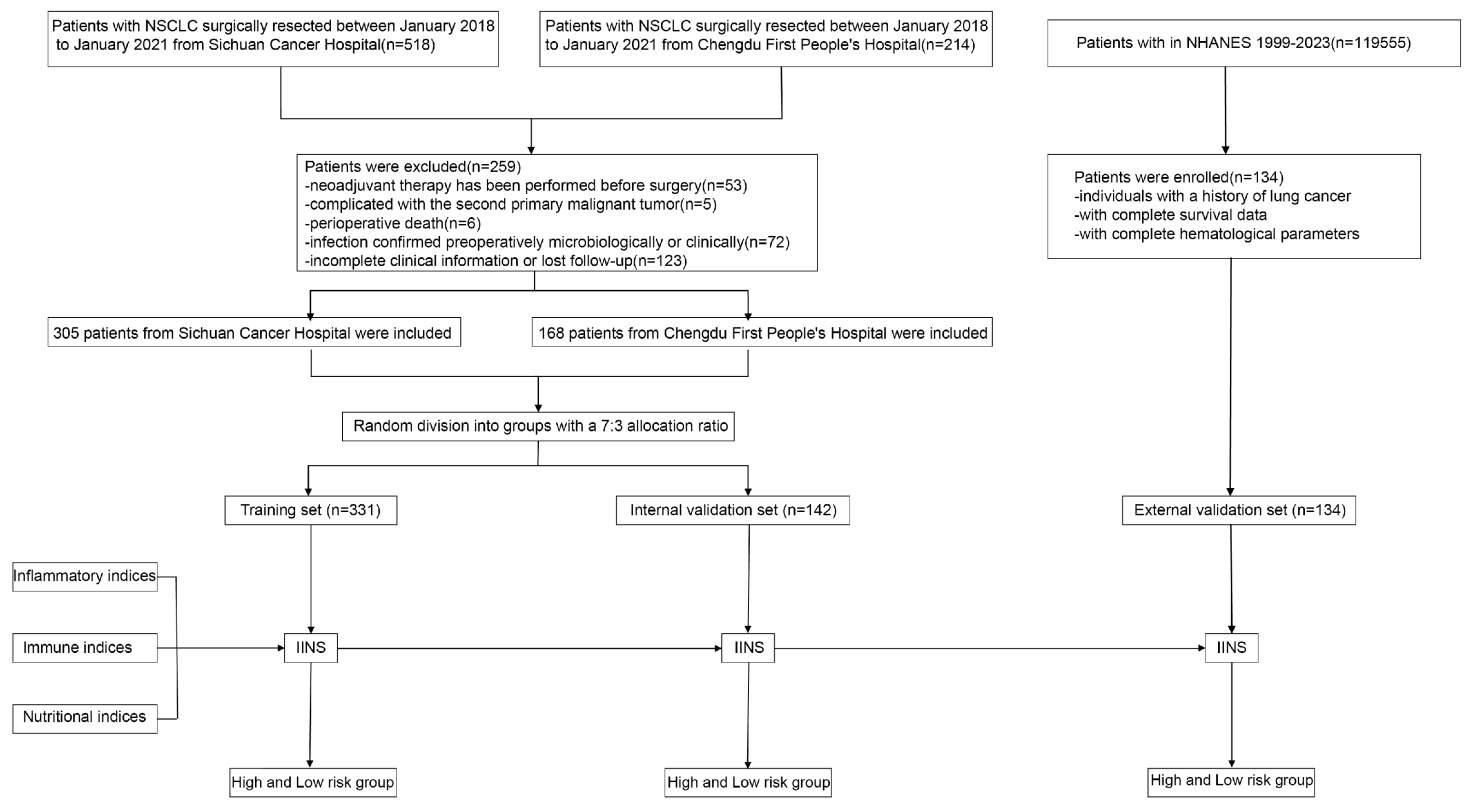
The overview of this study workflow from patient selection to statistical modeling and validation. A visual representation of the study design and data-processing pipeline.

For external validation, patients with a history of lung cancer were identified from the 1999–2023 National Health and Nutrition Examination Survey (NHANES) database (Ahluwalia et al., 2016). Only cases with complete demographic information, laboratory profiles, and survival follow-up data were included, resulting in an independent validation cohort of 134 individuals. This step ensured that the model’s performance could be evaluated across a geographically and ethnically diverse population. An overview of the study workflow, from patient selection to statistical modeling and validation, is illustrated in Fig. 1 to provide a visual representation of the study design and data-processing pipeline.

This study was reviewed and approved by the Ethics Committee for Medical Research and New Medical Technology of Sichuan Cancer Hospital, Chengdu, Sichuan, China (Approval No. SCCHEC-2022-118). The Institutional Review Board waived the requirement for informed consent because the study was retrospective in nature, and all patient data were anonymized prior to analysis.

### Surgical and perioperative management

Definitive surgical treatment consisted of complete (R0) tumor resection, carried out according to the National Comprehensive Cancer Network (NCCN) guidelines for NSCLC (version 4.2017). Depending on tumor location and extent, procedures included lobectomy, bilobectomy, or pneumonectomy, with every case accompanied by systematic mediastinal lymph node dissection to ensure accurate staging and clearance of microscopic disease. Following surgery, decisions regarding adjuvant chemotherapy, radiotherapy, or targeted therapy were made in accordance with NCCN recommendations, taking into account the patient’s pathological stage, overall performance status, and input from a multidisciplinary tumor board. All adverse events were documented and graded according to standardized reporting criteria. Perioperative care followed conventional protocols rather than enhanced recovery after surgery (ERAS) pathways. All patients were fasted for at least six hours before anesthesia induction. Those with preoperative insomnia were administered diazepam (10 mg intramuscularly or 1–2 mg orally) the night before surgery to reduce anxiety and improve sleep quality. After anesthesia induction, a urinary catheter was inserted and maintained until day three postoperatively. Analgesia was provided using patient-controlled intravenous infusion for 24–48 h, adjusted based on individual pain scores. Early mobilization and nutritional recovery were encouraged: patients were assisted to sit upright and begin a liquid diet on the first postoperative day, with progressive transition to oral feeding as tolerated. Ambulation was initiated as soon as clinically feasible to reduce the risk of complications such as deep venous thrombosis. Intravenous fluid therapy was maintained at 1,500–2,000 mL/day during the first three days, and thoracic drainage tubes were removed once the daily output fell below 100 mL. This comprehensive approach was designed to promote optimal recovery, minimize postoperative morbidity, and standardize patient management across both participating centers.

### Data collection

**Figure 2:**
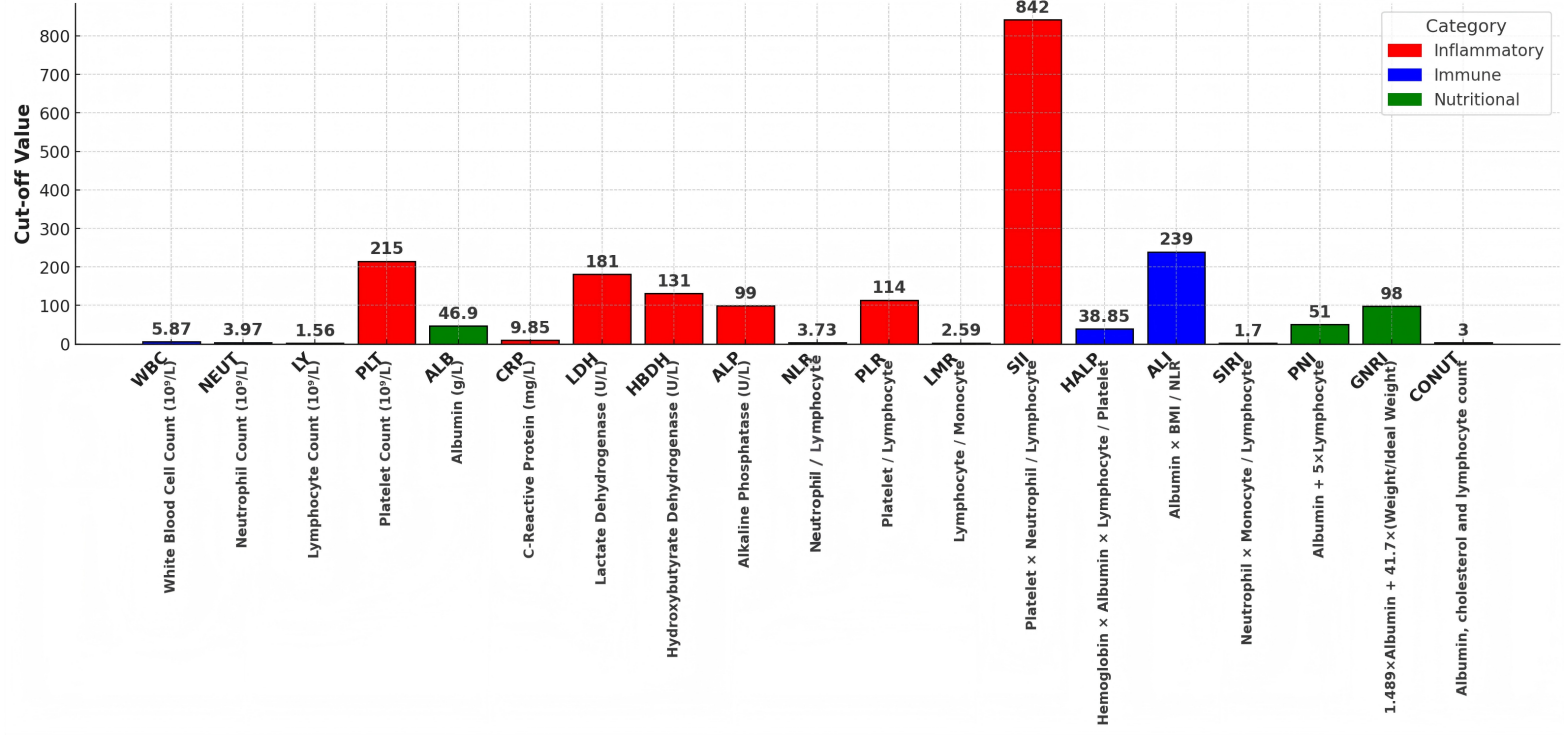
The calculation formulae and cut off value of inflammatory immune indicators and nutritional indicators. The CONUT score cutoff was set at 3, consistent with prior evidence. Detailed formulas and optimal cutoffs for all parameters.

All demographic and tumor-related characteristics of patients were collected, including age, gender, ECOG performance status, smoking history, resection type, histopathology, tumor diameter, differentiation, lymphovascular invasion, postoperative pathological stage, postoperative complications and adjuvant therapy. Preoperative hematological parameters within one week before surgery were collected *via* electronic medical record systems, including WBC count, NEUT count, lymphocyte count, platelet count, albumin, CRP, lactate dehydrogenase (LDH), hydroxybutyrate dehydrogenase (HBDH), alkaline phosphatase (ALP), and total serum cholesterol. Three categories of prognostic indices were calculated using internationally recognized consensus formulas: (1) Nutritional assessment indices: PNI, GNRI, and CONUT score, with the score graded levels (Naito et al., 2018); (2) Inflammation-related indices: NLR, PLR, LMR, and SII; (3) Composite biomarkers: Hemoglobin, Albumin, Lymphocyte, and Platelet (HALP) score, Advanced Lung Cancer Inflammation Index (ALI), SIRI, and albumin-to-alkaline phosphatase ratio (AAPR) .Optimal prognostic cutoff values for all continuous variables were determined using the Maximally Selected Rank Statistics method. The CONUT score cutoff was set at 3, consistent with prior evidence (Shao et al., 2021). Detailed formulas and optimal cutoffs for all parameters are provided in Fig. 2.

### Follow-up

This study employed a standardized tumor staging and follow-up system for survival analysis. Preoperative staging evaluation strictly adhered to the NCCN guidelines: (1) Contrast-enhanced chest CT to characterize the primary lesion; (2) Upper abdominal imaging (contrast-enhanced CT or ultrasonography) for abdominal metastasis assessment; (3) Contrast-enhanced brain MRI/CT to exclude cerebral metastasis. The postoperative surveillance protocol was similarly aligned with and implemented per NCCN guidelines: Follow-ups every 3 months within 2 years postoperatively, every 6 months during years 2–5, and annually thereafter. Standard surveillance comprised non-contrast chest CT, abdominal ultrasonography/CT, and laboratory tests (including complete blood count and hepatic/renal function panels). Suspected lesions triggered escalation to advanced imaging modalities (contrast-enhanced CT, MRI, or PET/CT). Follow-up monitoring concluded on 29 February 2024. The survival data for the external validation set were obtained from the NHANES 1999-2023 database.

### Development and validation of the IINS

This study applied a multi-phase modeling workflow to construct and verify the IINS prognostic scoring system. Initially, univariate Cox proportional hazards regression models were utilized to preliminarily identify potential predictors related to overall survival (OS). LASSO-Cox regression with 10-fold cross-validation was employed for further dimensionality reduction of candidate features. The optimal penalty coefficient (*λ*) was chosen to balance model complexity and goodness-of-fit, resulting in the IINS score as a linear combination of selected parameters. The predictive performance of IINS for OS and DFS was assessed using time-dependent ROC curves, with the AUC serving as the main metric. The Maximally Selected Rank Statistics method determined the optimal IINS cutoff value for OS discrimination, stratifying the cohort into high and low IINS groups. Kaplan–Meier analysis was used to assess survival differences between groups. For external validation, IINS scores were calculated using the predefined formula, and the optimal cutoff of 4.9 from the training cohort was applied to stratify patients. Survival disparities between risk groups in this independent cohort were evaluated *via* log-rank tests.

### Construction and validation of the prognostic model

Univariate Cox proportional hazards regression analysis was employed to screen for indicators significantly associated with prognosis. Indicators with statistically significant differences (*P* < 0.05) in the univariate analysis were included in a multivariate Cox proportional hazards model to determine independent prognostic factors. Based on the identified independent prognostic factors, a nomogram was constructed to predict DFS and OS. The model’s discriminative capacity was evaluated using ROC curves, along with AUC and concordance index (C-index) calculations. Calibration curves were used to assess the model’s calibration. Furthermore, DCA was applied to estimate the model’s clinical net benefit and examine its clinical utility.

### Statistical analysis

Statistical analyses were performed in R (v4.3.1). Group comparisons used Mann–Whitney U or chi-square tests as appropriate. Survival was estimated by Kaplan–Meier curves with log-rank tests. Model performance was assessed using C-index, ROC curves, decision curve analysis, and calibration plots. A *P* value < 0.05 was considered significant.

## Results

### Patient characteristics

A total of 473 individuals with NSCLC who underwent curative surgical resection were included, consisting of 301 men and 172 women, with a median age of 61 years (range: 22–82). During a median observation period of 50 months, 201 patients died and 231 experienced recurrence or metastasis. The dataset was randomly divided into a training cohort (331 cases) and a validation cohort (142 cases) using a 7:3 allocation ratio. The training group had a median follow-up of 51 months, with disease-free survival (DFS) rates at 1, 3, and 5 years of 76%, 56%, and 51%, and overall survival (OS) rates of 98%, 65%, and 58%. In comparison, the validation group had a median follow-up of 48.5 months, with DFS rates of 82%, 53%, and 49%, and OS rates of 94%, 66%, and 54% at the same time points.

No statistically significant differences in DFS or OS were noted between the two cohorts (*P* > 0.05). The baseline clinicopathological characteristics of patients in the training and validation cohorts are summarized in Table 1. As presented in Tables 1 and 2, baseline clinicopathological features and inflammation-, immunity-, and nutrition-related indicators were evenly distributed across groups, fulfilling the requirements for comparability in model evaluation.

**Table 1:** The baseline characteristics of patients with NSCLC in the training and validation set.

**Variables**		**Total (*n* = 473)**	**Training (*n* = 331)**	**Validation (*n* = 142)**	***P***
Gender	Female	172 (36.4%)	118 (35.7%)	54 (38.0%)	0.622
	Male	301 (63.6%)	213 (64.3%)	88 (62.0)	
Age (years)	≤65	306 (64.7%)	220 (66.5%)	86 (60.6%)	0.218
	>65	167 (35.3%)	111 (33.5%)	56 (39.4%)	
Smoking history	Non-smoker	212 (44.8%)	153 (46.2%)	59 (41.6%)	0.349
	Smoker	261 (55.2%)	178 (53.8%)	83 (58.4%)	
ECOG PS	0	172(36.4%)	127(38.4%)	45(31.7%)	0.166
	1	301(63.6%)	204(61.6%)	97(68.3%)	
FEV1/FVC	<70%	147(31.1%)	96(29.0%)	51(35.9%)	0.137
	≥70%	326(68.9%)	235(71.0%)	91(64.1%)	
Resection type	Lobectomy	343 (72.52%)	234 (70.69%)	109 (76.76%)	0.253
	Bilobectomy	99 (20.93%)	76 (22.96%)	23 (16.20%)	
	Pneumonectomy	31 (6.55%)	21 (6.34%)	10 (7.04%)	
Operation type	VATS	346 (73.1%)	239 (72.2%)	107 (75.3%)	0.479
	Thoracotomy	127(26.9%)	92 (27.8%)	35 (24.7%)	
Histopathology	Squamous cell	206 (43.6%)	137 (41.4%)	69 (48.6%)	0.286
	Adenocarcinoma	252 (53.2%)	182 (55.0)	70 (49.3%)	
	Adenosquamous	15 (3.2%)	12 (3.6%)	3 (2.1%)	
Tumor diameter	≤5 cm	254 (53.7%)	177 (53.5%)	77 (54.2%)	0.881
	>5 cm	219 (46.3%)	154 (46.5%)	65 (45.8%)	
Differentiation	Poor	315 (66.6%)	218 (65.9%)	97 (68.3%)	0.789
	Moderate	113 (23.9%)	82 (24.8%)	31 (21.8%)	
	Well	45 (9.5%)	31 (9.3%)	14 (9.9%)	
Lymphovascular invasion	No	222(46.9%)	156(47.1%)	66(46.5%)	0.897
	Yes	251(53.1%)	175(52.9%)	76(53.5%)	
Pleural invasion	No	195 (41.2%)	131 (39.6%)	64 (45.1%)	0.266
	Yes	278 (58.8%)	200 (60.4%)	78 (54.9%)	
Number of positive LNs	<4	333 (70.4%)	232 (70.1%)	101 (71.1%)	0.821
	≥4	140 (29.6%)	99 (29.9%)	41 (28.9%)	
Number of LNs dissected	<16	192 (40.6%)	133 (40.2%)	59 (41.5%)	0.781
	≥16	281 (59.4%)	198 (59.8%)	83 (58.5%)	
Postoperative complications	No	314(66.4%)	221(66.8%)	93(65.5%)	0.788
	Yes	159(33.6%)	110(33.2%)	49(34.5%)	
T stage	1	113 (23.9%)	83 (25.1%)	30 (21.1%)	0.617
	2	180 (38.1%)	124 (37.5%)	56 (39.5%)	
	3	71 (15.0%)	46 (13.9%)	25 (17.6%)	
	4	109 (23.0%)	78 (23.5%)	31 (21.8%)	
N stage	0	181 (38.3%)	127 (38.4%)	54 (38.0%)	0.346
	1	124 (26.2%)	81 (24.5%)	43 (30.3%)	
	2	168 (35.5%)	123 (37.1%)	45 (31.7%)	
TNM stage	1	118 (25.0%)	87 (26.3%)	31 (21.8%)	0.425
	2	108 (22.8%)	71 (21.5%)	37 (26.1%)	
	3	247 (52.2%)	173 (52.2%)	74 (52.1%)	

**Table 2:** The inflammatory immune and nutritional indicators between the training and validation set.

**Variables**	**Total (*n* = 473)**	**Training (*n* = 331)**	**Validation (*n* = 142)**	***Z***	***P***
WBC, M (Q_1_, Q_3_)	6.58 (5.33, 8.19)	6.65 (5.43, 8.29)	6.39 (5.22, 8.01)	−1.23	0.217
NEUT, M (Q_1_, Q_3_)	4.32 (3.27, 5.83)	4.40 (3.26, 5.97)	3.99 (3.31, 5.35)	−1.39	0.165
LY, M (Q_1_, Q_3_)	1.59 (1.25, 2.05)	1.62 (1.31, 2.08)	1.51 (1.18, 2.00)	−1.70	0.090
PLT, M (Q_1_, Q_3_)	191.0 (154, 242)	193.0 (155, 247)	190.0 (149, 233)	−1.17	0.241
ALB, M (Q_1_, Q_3_)	42.2 (39.0, 45.0)	42.4 (39.0, 45.0)	42.0 (39.0, 44.9)	−0.37	0.710
CRP, M (Q_1_, Q_3_)	5.02 (2.52, 8.00)	5.02 (2.89, 7.87)	5.02 (2.33, 8.01)	−0.92	0.360
LDH, M (Q_1_, Q_3_)	182.0 (157, 198)	181.0 (156, 197)	182.0 (161, 199)	−0.69	0.488
HBDH, M (Q_1_, Q_3_)	145.0 (127, 170)	145.0 (128, 171)	145.0 (125, 170)	−0.34	0.737
ALP, M (Q_1_, Q_3_)	103.0 (83, 119)	102.0 (83, 120)	106.0 (85, 119)	−0.76	0.448
NLR, M (Q_1_, Q_3_)	2.65 (1.90, 3.72)	2.65 (1.95, 3.72)	2.67 (1.82, 3.75)	−0.15	0.883
PLR, M (Q_1_, Q_3_)	118.0 (88, 163)	118.0 (88, 165)	127.0 (88, 160)	−0.43	0.670
LMR, M (Q_1_, Q_3_)	3.93 (2.85, 5.36)	4.03 (2.89, 5.35)	3.77 (2.70, 5.36)	−0.99	0.321
SII, M (Q_1_, Q_3_)	514.0 (328, 806)	532.0 (334, 809)	440.0 (320, 770)	−0.75	0.455
HALP, M (Q_1_, Q_3_)	47.3 (32.5, 65.5)	48.4 (32.5, 67.1)	44.1 (32.9, 63.8)	−0.61	0.541
ALI, M (Q_1_, Q_3_)	386.0(242, 543)	389.0 (242, 536)	383.0 (257, 544)	−0.05	0.964
SIRI, M (Q_1_, Q_3_)	1.06 (0.67, 1.72)	1.06 (0.67, 1.75)	1.05 (0.69, 1.66)	−0.01	0.989
PNI, M (Q_1_, Q_3_)	51.00 (47, 54)	51.00 (47, 54)	50.00 (47, 54)	−0.51	0.611
GNRI, M (Q_1_, Q_3_)	107 (101, 114.00)	107.0 (101, 114)	108.0 (101, 114)	−0.24	0.811
AAPR, M (Q_1_, Q_3_)	0.40 (0.35, 0.52)	0.40 (0.34, 0.53)	0.41 (0.35, 0.48)	−0.26	0.793

### Development and validation of the IINS

**Figure 3:**
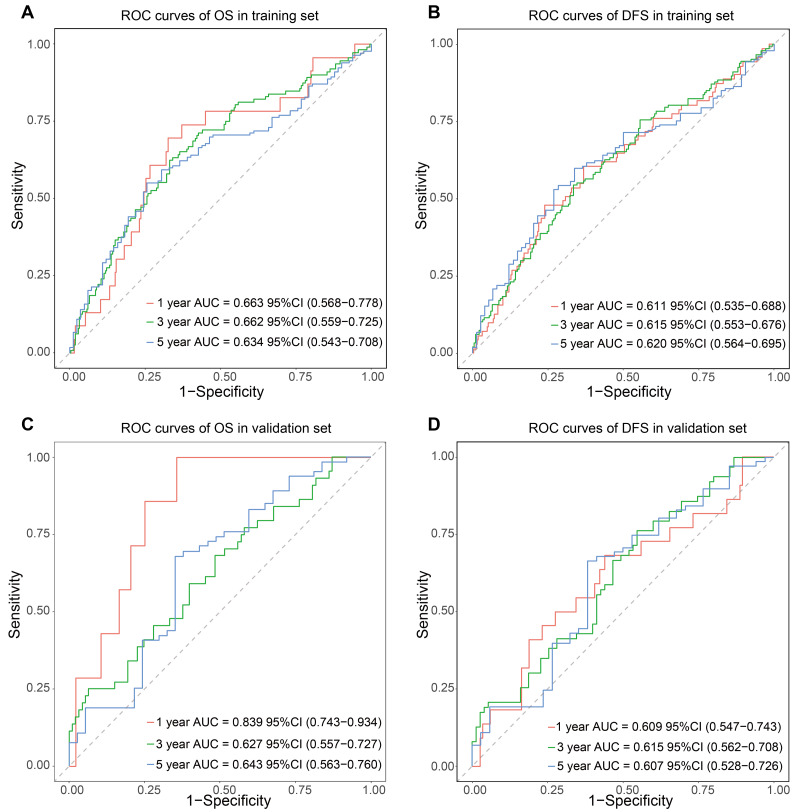
ROC curves of IINS for predicting DFS and OS in the training and validation sets.

**Figure 4:**
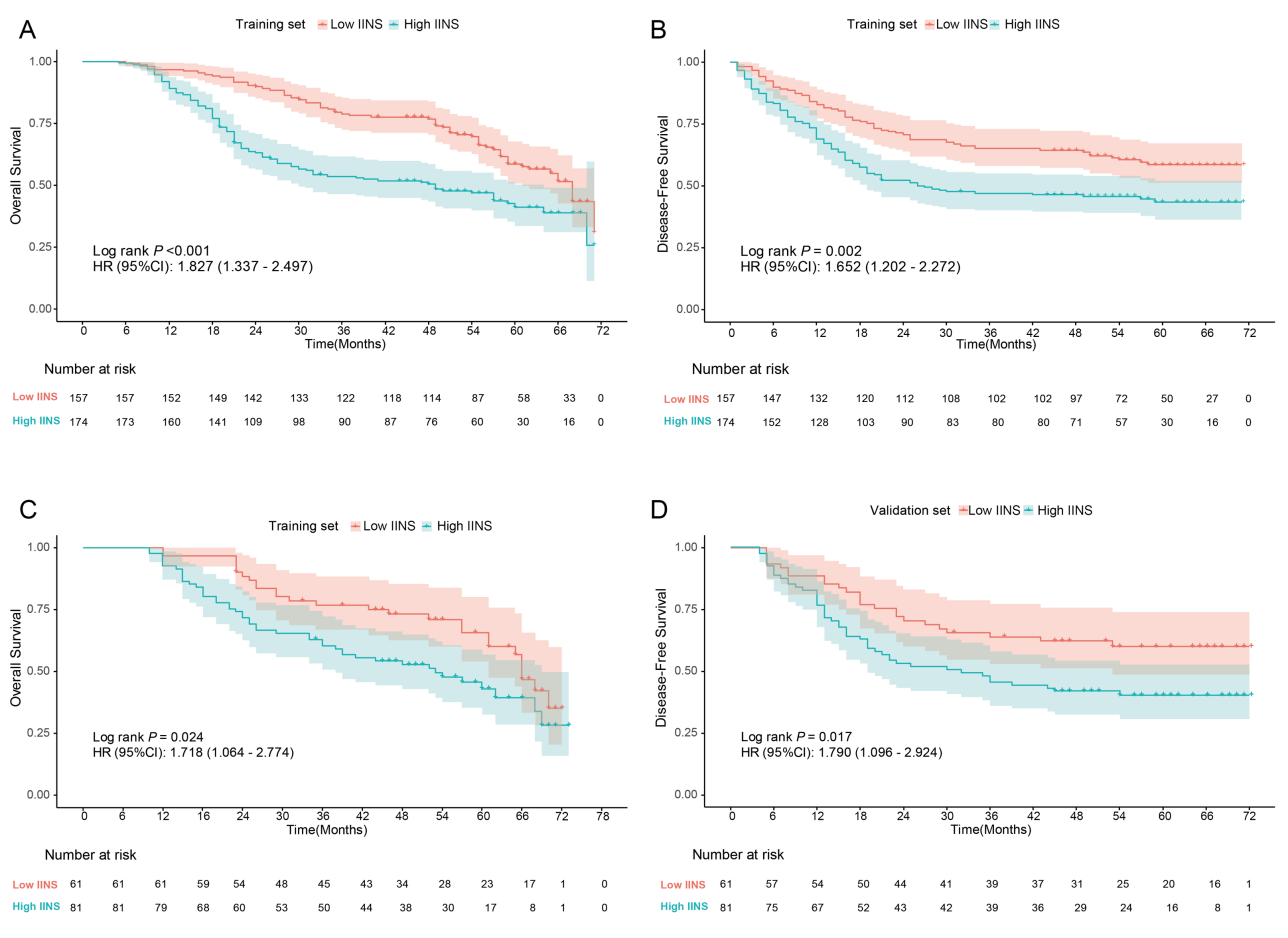
Kaplan–Meier survival curves stratified by IINS risk groups in the training (A–B) and validation (C–D) cohorts. Low-IINS and high-IINS groups were defined using the optimal IINS cut-off value.

Univariate Cox regression analysis identified 19 variables significantly associated with OS and 16 variables with DFS (*P* < 0.05, Table 3). Subsequently, the LASSO-COX regression algorithm combined with 10-fold cross-validation was applied for variable selection. The optimal regularization parameter *λ* was selected by minimizing deviance, retaining three variables with non-zero coefficients (PLR, SIRI, PNI) to establish the IINS prognostic scoring system (Fig. S1). The IINS formula was defined as: IINS = 0.10396001 × PLR + 0.04613632 × SIRI−0.14080046 × PNI. The optimal cutoff value for the IINS was identified as 4.9 based on the maximum selected rank statistic. Analysis of the relationship between IINS and clinicopathological characteristics showed significant associations with lymphovascular invasion (*P* = 0.011), number of dissected lymph nodes (*P* = 0.006), and T stage (*P* = 0.015), as detailed in Table 4. Time-dependent ROC analysis demonstrated stable predictive performance of IINS for OS and DFS, with AUC values > 0.60 in both training and validation cohorts (Fig. 3). Patients were stratified into high IINS (≥4.9) and low IINS (<4.9) groups based on the predefined cutoff. Kaplan–Meier analysis demonstrated that the high IINS group had significantly poorer DFS and OS compared to the low IINS group (*P* < 0.05, Fig. 4).

**Table 3:** Correlation analysis of inflammatory, immune and nutritional biomarkers with prognosis in non-small cell lung cancer.

**Variables**	**OS**		**DFS**	
	**HR (95% CI)**	***P***	**HR (95% CI)**	***P***
WBC (<5.87/≥5.87)	1.50 (1.14∼1.98)	0.004	1.41 (1.06∼1.86)	0.016
NEUT (<3.97/≥3.97)	1.34 (1.03∼1.74)	0.027	1.24 (0.95∼1.62)	0.107
LY (<1.56/≥1.56)	0.76 (0.59∼0.98)	0.031	0.71 (0.55∼0.92)	0.011
PLT (<215/≥215)	1.61 (1.25∼2.08)	<.001	1.47 (1.13∼1.91)	0.004
ALB (<46.9/≥46.9)	0.71 (0.50∼1.02)	0.062	0.63 (0.42∼0.93)	0.021
CRP (<9.85/≥9.85)	1.63 (1.20∼2.22)	0.002	1.91 (1.41∼2.59)	<.001
LDH (<181/≥181)	1.52 (1.17∼1.98)	0.001	1.55 (1.19∼2.02)	<.001
HBDH (<131/≥131)	1.64 (1.19∼2.24)	0.002	1.25 (0.92∼1.69)	0.158
ALP (<99/≥99)	1.52 (1.17∼1.99)	0.002	1.78 (1.35∼2.34)	<.001
NLR (<3.73/≥3.73)	1.72 (1.30∼2.28)	<.001	1.74 (1.32∼2.30)	<.001
PLR (<114/≥114)	1.71 (1.32∼2.22)	<.001	1.58 (1.21∼2.06)	<.001
LMR (<2.59/≥2.59)	0.56 (0.42∼0.76)	<.001	0.66 (0.48∼0.89)	0.007
SII (<842/≥842)	1.80 (1.35∼2.40)	<.001	1.75 (1.31∼2.33)	<.001
HALP (<38.85/≥38.85)	0.50 (0.39∼0.65)	<.001	0.55 (0.43∼0.72)	<.001
ALI (<239/≥239)	0.57 (0.43∼0.76)	<.001	0.59 (0.45∼0.79)	<.001
SIRI (<1.7/≥1.7)	1.60 (1.21∼2.10)	<.001	1.40 (1.05∼1.86)	0.021
PNI (<51/≥51)	0.65 (0.51∼0.84)	<.001	0.64 (0.49∼0.83)	<.001
GNRI (<98/≥98)	0.72 (0.53∼0.98)	0.036	0.85 (0.62∼1.18)	0.336
AAPR (<0.484/≥0.484)	0.64 (0.48∼0.86)	0.003	0.52 (0.37∼0.71)	<.001
CONUT (<3/≥3)	1.49 (1.10∼2.02)	0.010	1.71 (1.27∼2.30)	<.001

**Table 4:** Association of IINS with clinicopathological characteristics of NSCLC patients.

**Variables**		**Total** **(*N* = 473)**	**IINS** <** 4.9** **(*N* = 218)**	**IINS≥4.9** **(*N* = 255)**	***P***
Gender	Female	172 (36.4)	87 (18.4)	85 (18.0)	
	Male	301 (63.6)	131 (27.7)	170 (35.9)	0.138
Age (years)	≤65	306 (64.7)	150 (31.7)	156 (33.0)	
	>65	167 (35.3)	68 (14.4)	99 (20.9)	0.083
Smoking history	Non-smoker	212 (44.8)	98 (20.7)	114 (24.1)	
	smoker	261 (55.2)	120 (25.4)	141 (29.8)	0.957
ECOG PS	0	172 (36.36)	80 (36.70)	92 (36.08)	
	1	301 (63.64)	138 (63.30)	163 (63.92)	0.889
FEV1/FVC	<70%	147 (31.08)	64 (29.36)	83 (32.55)	
	≥70%	326 (68.92)	154 (70.64)	172 (67.45)	0.455
Histopathology	Squamous cell	206 (43.6)	95 (20.1)	111 (23.5)	
	Adenocarcinoma	252 (53.3)	118 (24.9)	134 (28.3)	0.595
	Adenosquamous	15 (3.1)	5 (1.1)	10 (2.1)	
Tumor diameter	≤5 cm	254 (53.7)	121 (25.6)	133 (28.1)	
	>5 cm	219 (46.3)	97 (20.5)	122 (25.8)	0.467
Differentiation	Poor	315 (66.6)	136 (28.8)	179 (37.8)	
	Moderate	113 (23.9)	61 (12.9)	52 (11.0)	0.141
	Well	45 (9.5)	21 (4.4)	24 (5.1)	
Lymphovascular invasion	No	222 (46.93)	116 (53.21)	106 (41.57)	
	Yes	251 (53.07)	102 (46.79)	149 (58.43)	0.011
Pleural invasion	No	195 (41.23)	94 (43.12)	101 (39.61)	
	Yes	278 (58.77)	124 (56.88)	154 (60.39)	0.439
Number of positive LNs	<4	333 (70.4)	151 (31.9)	182 (38.5)	
	≥4	140 (29.6)	67 (14.2)	73 (15.4)	0.617
Number of LNs dissected	<16	192 (40.6)	74 (15.6)	118 (24.9)	
	≥16	281 (59.4)	144 (30.4)	137 (29.0)	0.006
T stage	1	113 (23.9)	55 (11.6)	58 (12.3)	
	2	180 (38.1)	81 (17.1)	99 (20.9)	
	3	71 (15.0)	22 (4.7)	49 (10.4)	0.015
	4	109 (23.0)	60 (12.7)	49 (10.4)	
N stage	0	181 (38.3)	74 (15.6)	107 (22.6)	
	1	124 (26.2)	67 (14.2)	57 (12.1)	0.077
	2	168 (35.5)	77 (16.3)	91 (19.2)	
TNM stage	1	118 (25.0)	55 (11.6)	63 (13.3)	
	2	108 (22.8)	49 (10.4)	59 (12.5)	0.982
	3	247 (52.2)	114 (24.1)	133 (28.1)	

### Development of the prognostic model

This study systematically analysed prognostic factors in NSCLC patients using Cox regression models. Univariate analysis identified age, FEV1/FVC, resection type, number of positive lymph nodes, differentiation, lymph vascular invasion, T stage, N stage, and IINS as significant factors affecting OS (*P* < 0.05, Table 5). Factors such as gender, resection type, number of dissected lymph nodes, lymph vascular invasion, T stage, N stage, and IINS were significantly linked to DFS (*P* < 0.05, Table 6). Multivariate analysis identified age (HR = 1.57, 95% CI [1.07–2.29], *P* = 0.020), lymphovascular invasion (HR = 1.87, 95% CI [1.19–2.93], *P* = 0.007), N stage (HR = 3.03, 95% CI [1.79–5.11], *P* < 0.001), and IINS (HR = 1.63, 95% CI [1.17–2.26], *P* = 0.004) as independent risk factors for OS (Table 5). Independent prognostic factors for DFS included gender (HR = 1.51, 95% CI [1.03–2.20], *P* = 0.034), number of dissected lymph nodes (HR = 0.63, 95% CI [0.45–0.88], *P* = 0.007), lymphovascular invasion (HR = 1.65, 95% CI [1.09–2.50], *P* = 0.019), N stage (HR = 2.05, 95% CI [1.39–3.04], *P* < 0.001), and IINS (HR = 1.41, 95% CI [1.02–1.97], *P* = 0.039) (Table 6).

**Table 5:** Analyses of prognostic factors for OS in training cohort.

**Variables**		**Univariable analysis**		**Multivariable analysis**	
		**HR (95% CI)**	***P***	**HR (95% CI)**	***P***
Gender	Female	1.00			
	Male	1.16 (0.84∼1.60)	0.356		
Age (years)	≤65	1.00		1.00	
	>65	1.56 (1.14∼2.13)	0.005	1.57 (1.07∼2.29)	0.020
Smoking history	Non-smoker	1.00			
	Smoker	1.25 (0.92∼1.71)	0.154		
ECOG PS	0	1.00			
	1	1.15 (0.84∼1.58)	0.388		
FEV1/FVC	<70%	1.00		1.00	
	≥70%	0.71(0.52∼0.98)	0.035	0.93 (0.62∼1.39)	0.724
Resection type	Lobectomy	1.00		1.00	
	Bilobectomy	1.02 (0.71∼1.46)	0.903	1.18 (0.80∼1.73)	0.409
	Pneumonectomy	1.97 (1.10∼3.50)	0.022	1.62 (0.88∼2.99)	0.119
Operation type	VATS	1			
	Thoracotomy	1.03(0.71∼1.49)	0.868		
Number of positive LNs	<4	1.00		1.00	
	≥4	1.52 (1.11∼2.09)	0.009	1.32 (0.84∼2.08)	0.205
Number of LNs dissected	<16	1.00			
	≥16	0.88 (0.65∼1.20)	0.424		
Tumor diameter	≤5 cm	1.00			
	>5 cm	1.06 (0.78∼1.45)	0.688		
Differentiation	Poor	1.00		1.00	
	Moderate	0.84 (0.50∼1.42)	0.511	0.96 (0.57∼1.69)	0.668
	Well	0.67 (0.46∼0.99)	0.044	0.90 (0.61∼1.35)	0.938
Lymphovascular invasion	No	1.00		1.00	
	Yes	1.79 (1.31∼2.46)	<.001	1.87 (1.19∼2.93)	0.007
Pleural invasion	No	1.00			
	Yes	1.29 (0.94∼1.77)	0.120		
Histopathology	Squamous cell	1.00			
	Adenocarcinoma	0.96 (0.70∼1.32)	0.819		
	Adenosquamous	0.79 (0.32∼1.97)	0.619		
T stage	1	1.00		1.00	
	2	1.41 (0.88∼2.24)	0.152	1.02 (0.62∼1.66)	0.919
	3	1.68 (1.09∼2.58)	0.019	1.16 (0.60∼2.27)	0.626
	4	2.09 (1.26∼3.47)	0.004	1.89 (0.98∼3.70)	0.051
N stage	0	1.00		1.00	
	1	1.34 (0.88∼2.06)	0.174	1.61 (1.01∼2.57)	0.046
	2	2.26 (1.57∼3.26)	<.001	3.03 (1.79∼5.11)	<.001
Adjuvant radiotherapy	No	1.00			
	Yes	1.34 (0.99∼1.82)	0.061		
Adjuvant chemotherapy	No	1.00			
	Yes	0.86 (0.63∼1.16)	0.324		
IINS	<4.9	1.00		1.00	
	≥4.9	1.83 (1.34∼2.50)	<.001	1.63 (1.17∼2.26)	0.004
Postoperative complications	No	1.00			
	Yes	1.09(0.79∼1.51)	0.592		

**Table 6:** Analyses of prognostic factors for DFS in training cohort.

**Variables**		**Univariable analysis**		**Multivariable analysis**	
		**HR (95% CI)**	***P***	**HR (95% CI)**	***P***
Gender	Female	1.00		1.00	
	Male	1.82 (1.28∼2.60)	<.001	1.51 (1.03∼2.20)	0.034
Age (years)	≤65	1.00			
	>65	1.28 (0.93∼1.77)	0.128		
Smoking history	Non-smoker	1.00			
	Smoker	1.29 (0.94∼1.77)	0.114		
ECOG PS	0	1.00			
	1	1.17 (0.85∼1.62)	0.332		
FEV1/FVC	<70%	1.00			
	≥70%	0.92 (0.65∼1.29)	0.615		
Resection type	Lobectomy	1.00		1.00	
	Bilobectomy	0.84 (0.57∼1.25)	0.392	0.98 (0.64∼1.49)	0.920
	Pneumonectomy	1.82 (1.06∼3.12)	0.029	1.52 (0.85∼2.70)	0.154
Operation type	VATS	1			
	Thoracotomy	1.05(0.73∼1.49)	0.802		
Number of positive LNs	<4	1.00			
	≥4	1.04 (0.74∼1.46)	0.817		
Number of LNs dissected	<16	1.00		1.00	
	≥16	0.72 (0.53∼0.99)	0.042	0.63 (0.45∼0.88)	0.007
Tumor diameter	≤5 cm	1.00			
	>5 cm	1.12 (0.82∼1.54)	0.467		
Differentiation	Poor	1.00			
	Moderate	0.77 (0.53∼1.12)	0.177		
	Well	0.58 (0.31∼1.08)	0.088		
Lymphovascular invasion	No	1.00		1.00	
	Yes	1.47 (1.08∼2.02)	0.014	1.65 (1.09∼2.50)	0.019
Pleural invasion	No	1.00			
	Yes	1.06 (0.77∼1.46)	0.711		
Histopathology	Squamous cell	1.00			
	Adenocarcinoma	1.02 (0.74∼1.40)	0.925		
	Adenosquamous	1.13 (0.49∼2.62)	0.776		
T stage	1	1.00		1.00	
	2	1.09 (0.66∼1.81)	0.729	1.16 (0.60∼2.22)	0.647
	3	1.84 (1.09∼3.11)	0.021	1.30 (0.81∼2.08)	0.276
	4	1.90 (1.25∼2.90)	0.003	1.56 (0.82∼2.94)	0.101
N stage	0	1.00		1.00	
	1	1.03 (0.66∼1.59)	0.857	1.19 (0.75∼1.89)	0.452
	2	1.76 (1.22∼2.49)	0.002	2.05 (1.39∼3.04)	<.001
Adjuvant radiotherapy	No	1.00			
	Yes	1.03 (0.76∼1.41)	0.836		
Adjuvant chemotherapy	No	1.00			
	Yes	0.77 (0.57∼1.06)	0.112		
IINS	0	1.00		1.00	
	1	1.65 (1.21∼2.29)	0.002	1.41 (1.02∼1.97)	0.039
Postoperative complications	No	1.00			
	Yes	1.14 (0.81∼1.59)	0.458		

**Figure 5:**
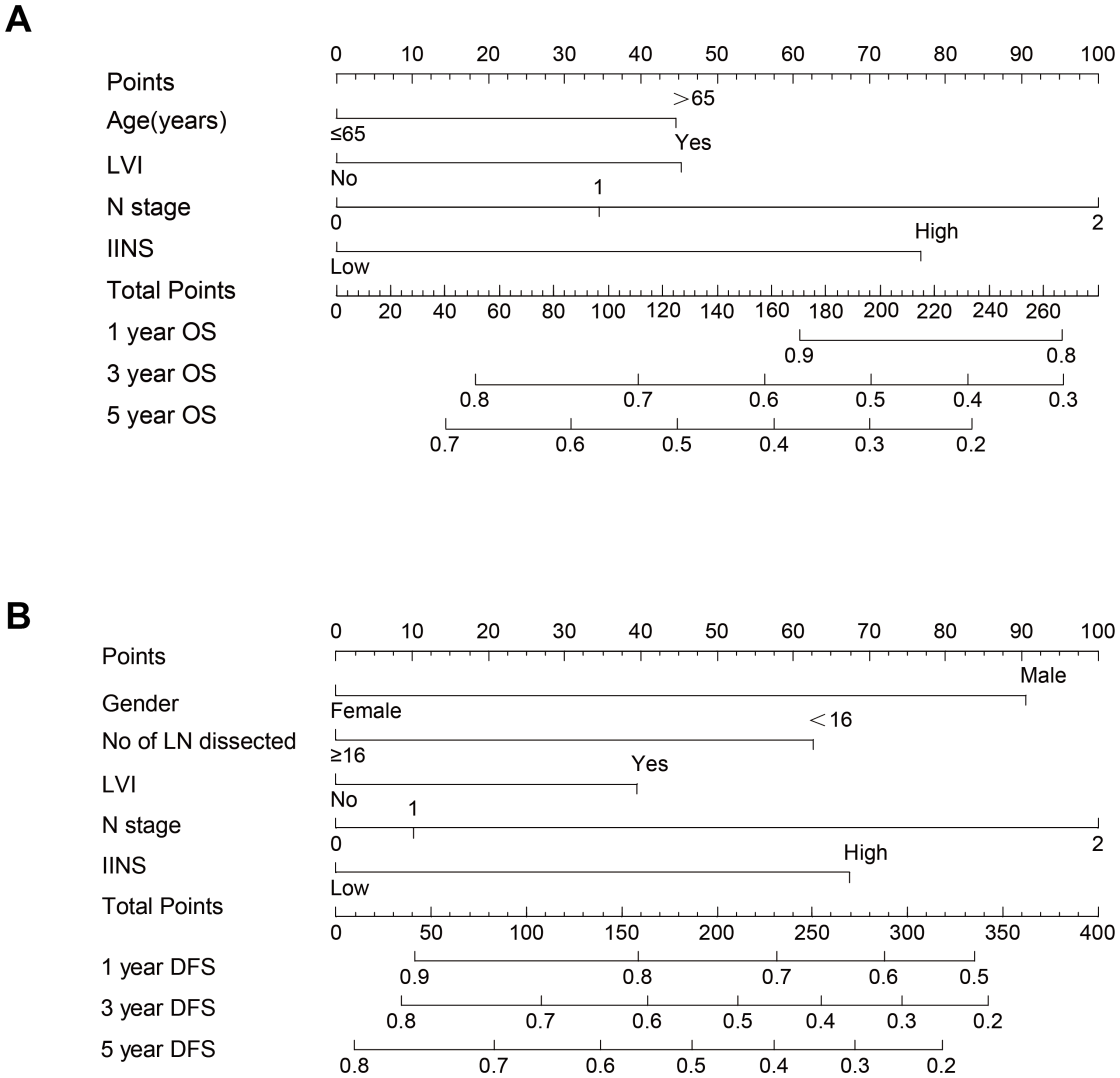
Prognostic nomograms (A) OS nomogram incorporating age,LVI, N stage, and IINS risk score. (B) DFS nomogram including gender, number of lymph nodes dissected, LVI, and IINS risk score.

**Figure 6:**
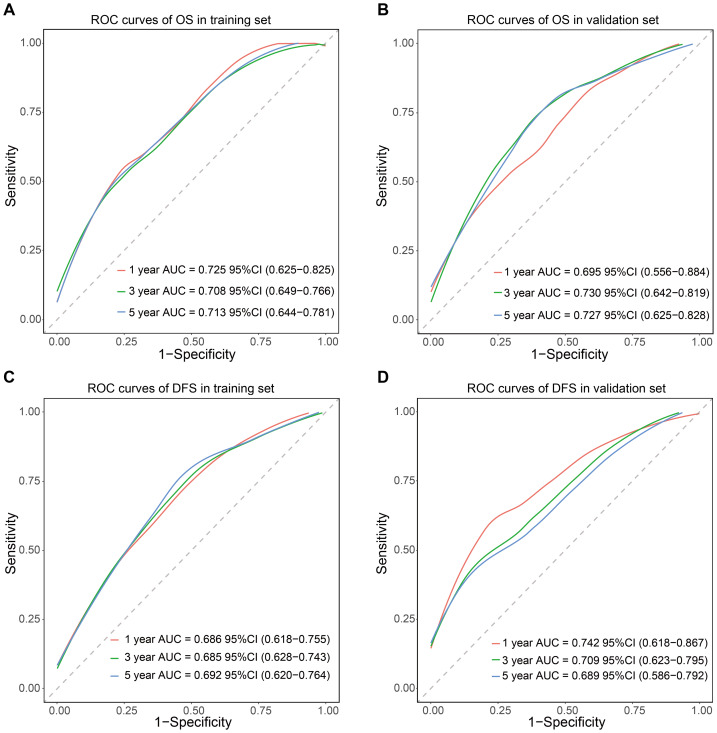
Time-dependent ROC analysis of the nomogram’s predictive performance for OS (A, B) and DFS (C, D) in the training and validation cohorts.

**Figure 7:**
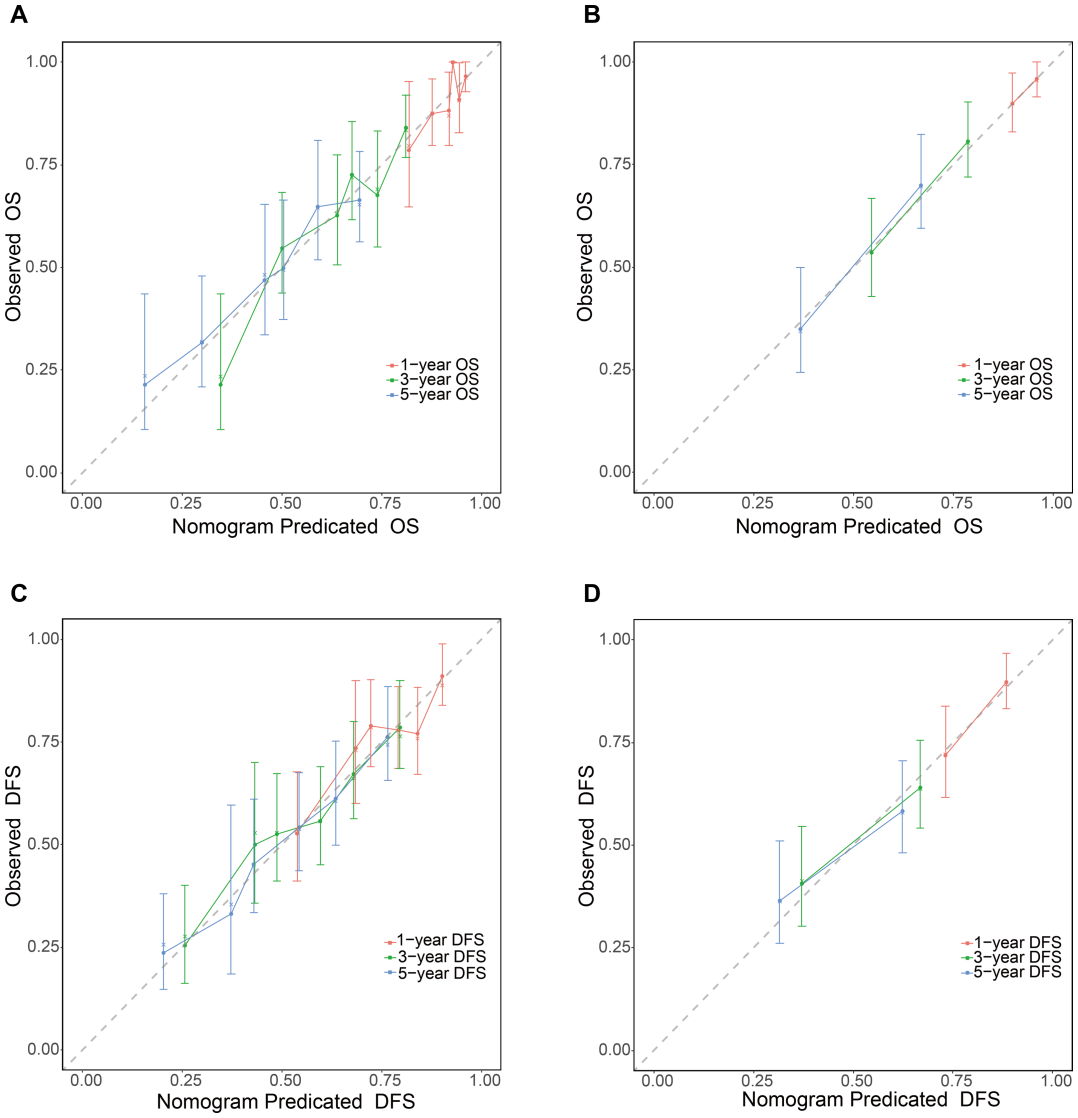
Calibration curves of the nomogram. The plots demonstrate the concordance between nomogram-predicted and observed probabilities of (A–B) OS and (C–D) DFS at 1, 3, and 5 years in the training and validation sets.

**Figure 8:**
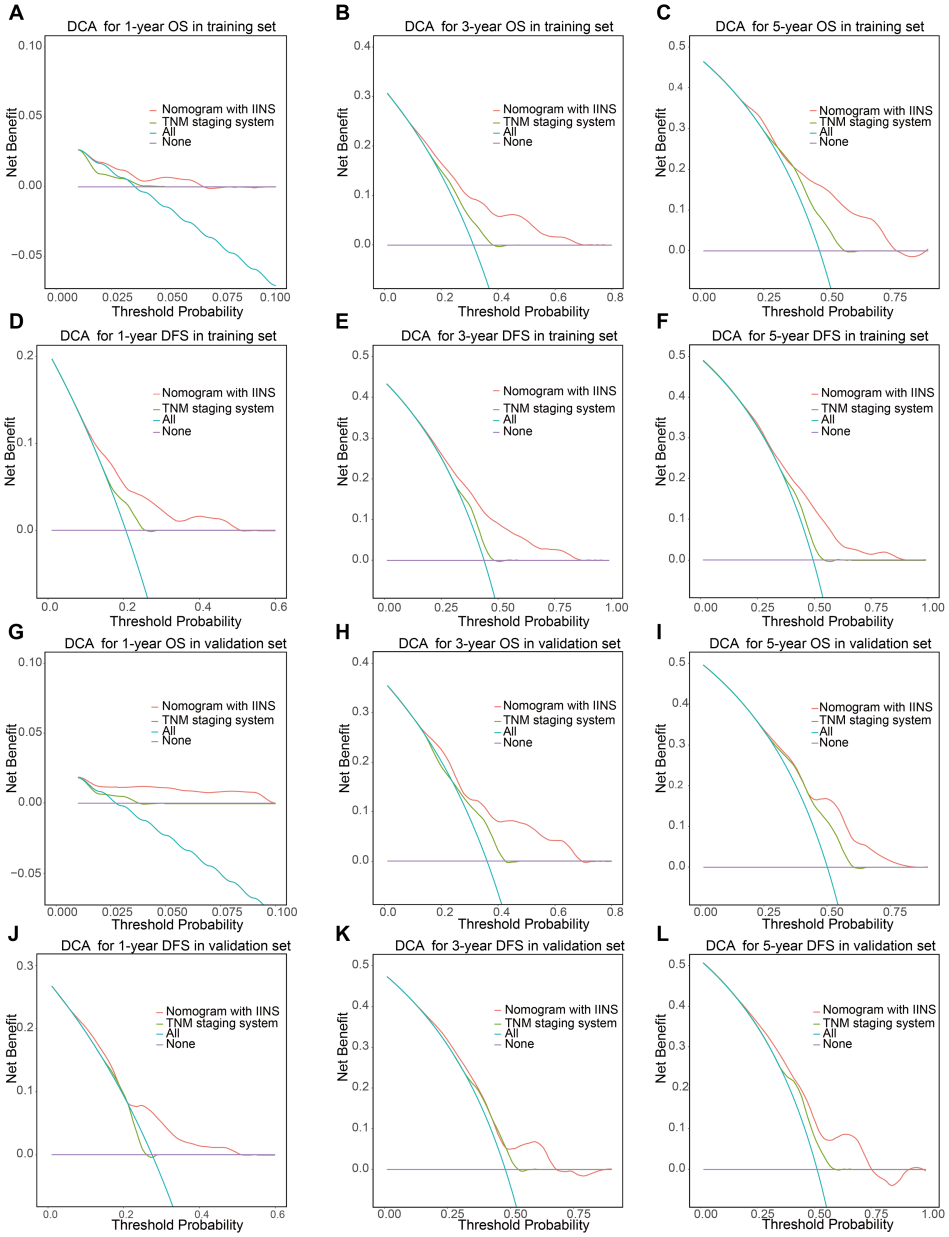
Decision curve analysis evaluating the clinical utility of the nomogram. The figure compares the net benefit of the nomogram (incorporating IINS) and the TNM system for predicting 1-, 3-, and 5-year OS and DFS in the training and validation sets.

The prognostic nomogram incorporating these independent predictors demonstrated favorable clinical utility (Fig. 5). ROC curve analysis demonstrated consistent predictive performance, with AUC values for 1-, 3-, and 5-year OS ranging from 0.708 to 0.725 in the training set and 0.695 to 0.730 in the validation set. Corresponding AUC values for DFS were 0.685 to 0.692 and 0.689 to 0.742, respectively (Fig. 6). Calibration curves showed high concordance between the nomogram-predicted probabilities and actual observations across all time points (Fig. 7). Decision curve analysis showed that the nomogram with IINS offered greater net clinical benefit than the traditional TNM staging system for predicting 1-, 3-, and 5-year OS and DFS in both training and validation cohorts (Fig. 8).

### External validation of the IINS

**Figure 9:**
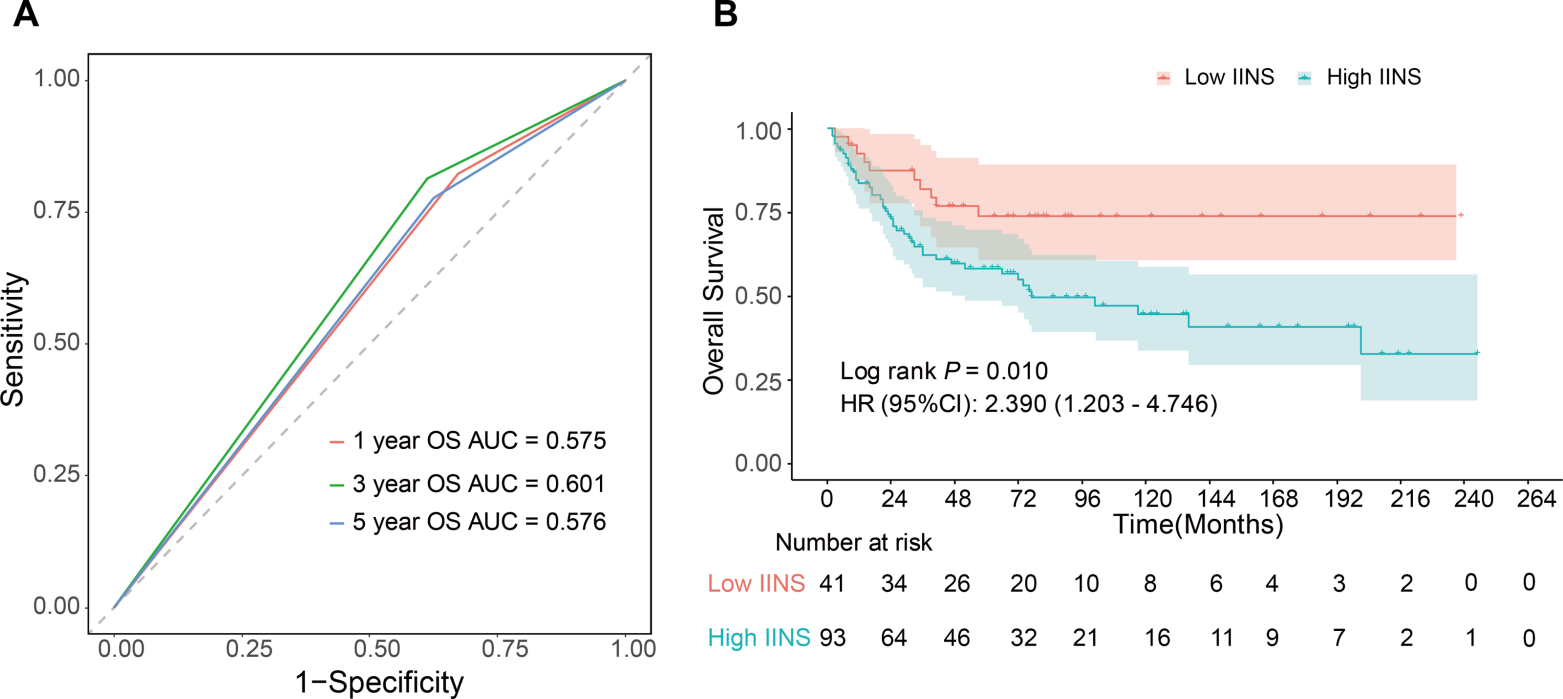
External validation of the IINS in the NHANES cohort. (A) Time-dependent ROC curves demonstrating 1-, 3-, and 5-year OS prediction accuracy. (B) Kaplan–Meier survival curves comparing IINS-defined high IINS *versus* low IINS groups.

This study externally validated the prognostic value of the IINS using the NHANES database (1999–2023). After screening 119,555 participants, 134 patients with a history of lung cancer and complete clinical and hematological data were included. The IINS was calculated for all patients using the predefined formula, and patients were stratified into high- and low-risk groups using the cutoff value of 4.9 established in the training cohort. Time-dependent ROC analysis demonstrated AUC values of 0.575–0.601 for predicting 1-, 3-, and 5-year OS (Fig. 9A), indicating modest discriminative ability in this heterogeneous external cohort. Survival analysis revealed significantly worse OS in the high IINS group (Fig. 9B). The validation cohort encompassed diverse racial groups (White, African American, Asian, etc.) across the United States. The IINS showed modest discriminative ability (AUC: 0.575–0.601) but effectively stratified patients into high- and low-risk groups with significantly different survival outcomes (*P* < 0.05).

## Discussion

This study established the IINS by integrating systemic inflammatory, immune, and nutritional indicators, and further developed a postoperative survival prediction nomogram model for NSCLC combined with clinicopathological features. Based on multi-center retrospective cohorts and the NHANES external validation cohort, the IINS demonstrated stable cross-cohort performance in predicting OS and DFS. Univariate analysis identified age, FEV1/FVC, resection type, number of positive lymph nodes, differentiation, lymphovascular invasion, T stage, N stage and IINS as significant factors influencing OS, while gender, resection type, number of dissected lymph nodes, lymphovascular invasion, T stage, N stage, and IINS were closely associated with DFS. Multivariate analysis confirmed age, lymphovascular invasion, N stage and IINS as independent risk factors for OS, and gender, number of dissected lymph nodes, lymphovascular invasion, N stage and IINS as independent predictors for DFS. The nomogram model exhibited robust predictive performance in both training and validation cohorts, with 1-, 3-, and 5-year AUC values for OS reaching 0.725, 0.708, and 0.713, and for DFS achieving 0.686, 0.685, and 0.692, respectively, significantly outperforming traditional TNM staging systems. Although the discriminative performance of the IINS and the IINS-based nomogram, with AUC values ranging from approximately 0.60 to 0.71, may be considered moderate, this level of discrimination is consistent with that reported for several recently published prognostic models in oncology. For example, contemporary cancer prognostic models integrating clinical or laboratory variables have reported comparable AUC ranges in postoperative or real-world cohorts, where biological heterogeneity and treatment variability inherently limit maximal discrimination. Importantly, AUC alone does not fully capture clinical utility. The added value of IINS lies in its ability to complement TNM staging by incorporating host-related biological information, including systemic inflammation, immune status, and nutritional reserve, which are not represented in anatomical classification systems. This complementary effect was further substantiated by decision curve analysis, which demonstrated superior net benefit for the IINS-based nomogram compared with TNM staging alone across clinically relevant threshold probabilities. The optimal IINS cutoff of 4.9, determined *via* the maximum selected rank statistic, stratified patients into high- and low IINS groups, with the high IINS group demonstrating consistently inferior OS and DFS across both internal and external validation cohorts. These findings not only provide a novel tool for NSCLC prognosis assessment but also offer critical insights into the interplay of inflammation, immunity, and nutrition within the tumor microenvironment. Notably, the primary clinical strength of the IINS does not lie in achieving exceptionally high discrimination, but in its simplicity, accessibility, and cost-effectiveness. Because the score is derived entirely from routinely obtained preoperative blood parameters, it can be readily implemented in daily clinical practice without additional testing, financial burden, or specialized infrastructure. This pragmatic advantage is particularly relevant in real-world and resource-limited settings.

From a methodological perspective, the incremental prognostic value of a novel biomarker can be demonstrated through several complementary approaches. In the present study, additive value was supported by three key findings: (i) IINS remained an independent prognostic factor in multivariable Cox models adjusted for established clinical predictors; (ii) nomograms incorporating IINS achieved higher discrimination than conventional staging alone; and (iii) decision curve analysis demonstrated superior net benefit across clinically relevant thresholds. While formal likelihood ratio testing or changes in the C-index may offer additional statistical quantification, the consistent improvement observed across discrimination, calibration, and clinical utility metrics collectively supports the additive prognostic contribution of IINS.

The complex regulatory network of the tumor microenvironment is centrally characterized by dynamic interactions among inflammatory responses, immune status, and nutritional metabolism (Quail & Joyce, 2013; Hanahan & Weinberg, 2011; Elia & Haigis, 2021). From a clinical and biological perspective, the structure of the IINS formula reflects the integrated burden of systemic inflammation, immune dysregulation, and nutritional impairment. Higher PLR and SIRI values contribute positively to the IINS score, indicating heightened inflammatory activity and suppressed antitumor immune surveillance, whereas PNI carries a negative coefficient, such that lower nutritional status results in a higher overall IINS score. Consequently, a higher IINS biologically represents a state characterized by excessive systemic inflammation, immune dysfunction, and compromised nutritional reserve, conditions that collectively promote tumor progression, metastasis, and resistance to treatment. Tumor-associated inflammation promotes immunosuppressive cell infiltration through activation of the NF-κB/STAT3 pathway, while inflammatory mediators directly alter metabolic states within the microenvironment, suppressing antitumor immune responses (Diakos et al., 2014). Nutritional signals (*e.g.*, glucose, glutamine) regulate histone acetylation to remodel chromatin accessibility, thereby determining the differentiation fate and functional impairment of exhausted CD8+ T cells (Ma et al., 2025). This multidimensional interplay not only drives tumor proliferation and metastasis but also reshapes the physical properties of the tumor microenvironment by altering stromal cell phenotypes, ultimately leading to therapeutic resistance and poor prognosis. The IINS was constructed using the selecting PLR, SIRI, and PNI as core parameters from 20 inflammation-, immune-, and nutrition-related indicators. Elevated PLR reflects not only enhanced platelet-mediated angiogenesis but also impaired immune surveillance due to lymphocytopenia, consistent with conclusions from prior studies (Templeton et al., 2014; Diem et al., 2017). A critical indicator of immune-inflammatory crosstalk in the tumor microenvironment and has been validated as a prognostic marker for multiple solid tumors (Tang et al., 2024; Qi et al., 2016; Chen et al., 2019). Reduced PNI is directly associated with decreased serum albumin levels, where hypoalbuminemia compromises immune function and anti-tumor responsiveness, establishing a malnutrition-immunosuppression vicious cycle (Arends et al., 2017; Seebacher et al., 2013; Xia et al., 2021). The synergistic effects of PLR, SIRI, and PNI collectively contribute to poor prognosis by promoting tumor proliferation, metastasis, and compromising host antitumor immunity. Compared with previous studies focusing on single biomarkers, the multidimensional integrative nature of the IINS enables more comprehensive capture of the heterogeneity in tumor biological behaviors, thereby partially explaining its superior predictive performance over traditional TNM staging systems.

Building upon these findings, the present study underscores the value of integrating systemic host factors into prognostic modeling for NSCLC. Unlike purely tumor-centric approaches, which rely heavily on genomic alterations or anatomical staging, the IINS provides a dynamic reflection of the patient’s immunologic and metabolic state, which can fluctuate in response to disease progression and treatment interventions. This holistic representation of the tumor-host interaction allows for earlier identification of patients at elevated risk, potentially before radiological or pathological progression becomes apparent. Furthermore, the ability of IINS to predict outcomes across multiple time points highlights its potential utility as a longitudinal monitoring tool. In clinical practice, such a score could be recalculated at defined intervals, enabling real-time risk adjustment and personalized modification of treatment plans, such as intensification of adjuvant therapy or initiation of nutritional and anti-inflammatory interventions. These attributes position the IINS as more than a static risk classifier, it may evolve into a cornerstone biomarker for adaptive clinical decision-making in the era of precision oncology.

This study examined the correlation between IINS and clinical characteristics. Patients with high IINS scores exhibited more aggressive clinicopathological features, including a more advanced T stage, increased number of dissected lymph nodes, and a higher incidence of lymphovascular invasion. These findings indicate that elevated IINS is associated with a more aggressive tumor phenotype, which may contribute to the poorer prognosis observed in these patients. Such features are well-established risk factors for residual occult micrometastases and locoregional recurrence. Integrating IINS with conventional staging parameters allows for refined stratification of biological aggressiveness, thereby improving the identification of patients at increased risk of postoperative recurrence and reduced survival. This underscores the clinical utility of IINS as a complementary tool that enhances prognostic assessment beyond conventional anatomic staging.

External validation in the multiethnic NHANES cohort (*n* = 134) demonstrated modest discriminative ability (AUC: 0.575−0.601), likely due to limited sample size and absence of detailed treatment data. Nevertheless, significant survival differences between risk groups support IINS as a stratification tool in diverse populations. The relatively modest AUC values observed in the NHANES external validation cohort are likely attributable to factors such as limited sample size, population heterogeneity, and the absence of detailed treatment-related variables in the database. Despite these limitations, the IINS retained its ability to significantly differentiate survival outcomes between high- and low-risk groups, supporting its utility as a stratification tool even in heterogeneous real-world populations. Furthermore, DCA revealed that integrating the IINS into clinical decision-making significantly enhanced net benefit rates for both overall survival (OS) and disease-free survival (DFS), outperforming the TNM staging system. These findings underscore the value of the IINS as a cost-effective and practical tool for risk assessment in clinical practice.

However, this study has several limitations that warrant consideration. First, the retrospective design may introduce selection bias, particularly with respect to postoperative treatment allocation and follow-up intensity. Second, although an independent external validation was performed using the NHANES database, the external cohort size was relatively small (*n* = 134), which may limit statistical power and preclude more detailed subgroup analyses. In addition, the NHANES database lacks granular oncologic treatment information, including surgical approach, chemotherapy regimens, radiotherapy dose, and immunotherapy exposure, which may introduce residual confounding when interpreting survival outcomes. These constraints are inherent to population-based databases and should be considered when extrapolating the external validation results. Finally, the IINS does not incorporate emerging molecular or immunologic biomarkers, such as PD-L1 expression or genomic alterations, which may further refine prognostic stratification in the era of precision oncology. Future prospective, multicenter studies with comprehensive treatment and molecular data are warranted to validate and extend the clinical applicability of the IINS.

## Conclusions

In conclusion, the IINS scoring system provides a reliable multidimensional tool for postoperative prognostic assessment in NSCLC by integrating inflammatory, immune, and nutritional indicators. The risk stratification based on IINS effectively complements the TNM staging system by quantifying host microenvironmental features, thereby providing a more comprehensive biological basis for individualized prognostic assessment and therapeutic strategy. Cross-cohort validation and clinical translatability demonstrate that the IINS effectively addresses the limitations of the TNM staging system in evaluating tumor biological heterogeneity by quantifying host microenvironment features. Through future prospective studies and mechanistic investigations, the IINS is poised to serve as a cornerstone for individualized therapeutic strategies, advancing the development of precision medicine.

## Supplemental Information

10.7717/peerj.21122/supp-1 Supplemental Information 1 Hematological Data of 473 NSCLC Patients Used for IINS Development and Validation The full de-identified dataset (n = 473) used to develop and validate the Inflammatory-Immune-Nutritional Score (IINS) for postoperative prognostic assessment in non-small cell lung cancer (NSCLC).

10.7717/peerj.21122/supp-2 Supplemental Information 2 LASSO Cox Regression for Variable Selection in IINS Model Development (A) Coefficient profiles of 20 candidate prognostic variables plotted against log(*λ*). Each colored line represents the trajectory of a variable’s coefficient as the regularization parameter changes. (B) Ten-fold cross-validation curve showing partial likelihood deviance versus log(*λ*). The left dashed line indicates the minimum deviance *λ*, and the right dashed line indicates the largest *λ* within 1-SE of the minimum, resulting in three non-zero coefficients (PLR, SIRI, PNI) retained for model construction.

10.7717/peerj.21122/supp-3 Supplemental Information 3 Explanation of original data encoding

## Additional Information and Declarations

### Data Availability

The following information was supplied regarding data availability: The full de-identified dataset of 473 NSCLC patients, including demographic variables (age, gender, smoking history, ECOG, BMI, FEV1/FVC), clinicopathological data (tumor diameter, histopathology, differentiation, LVI, PI, number of positive and dissected lymph nodes, pT/pN/pM stage, resection and operation type, postoperative complications, adjuvant radiotherapy and chemotherapy), preoperative hematological and biochemical parameters (WBC, NEUT, LY, PLT, MOMO, HGB, ALB, CRP, CHO, LDH, HBDH, ALP), calculated indices (NLR, PLR, LMR, SII, HALP, ALI, SIRI, CAR, PNI, GNRI, AAPR, CONUT), and outcome variables (DFS time and OS time in months) are available in the Supplemental File.
