# Supplementary figures and images for: Development and validation of an inflammatory-immune-nutritional integrated scoring system: a novel strategy for predicting postoperative survival in non-small cell lung cancer
Source: PeerJ. 2026 Apr 6;14:e21122. doi: 10.7717/peerj.21122 (PMC13064673; doi:10.7717/peerj.21122)

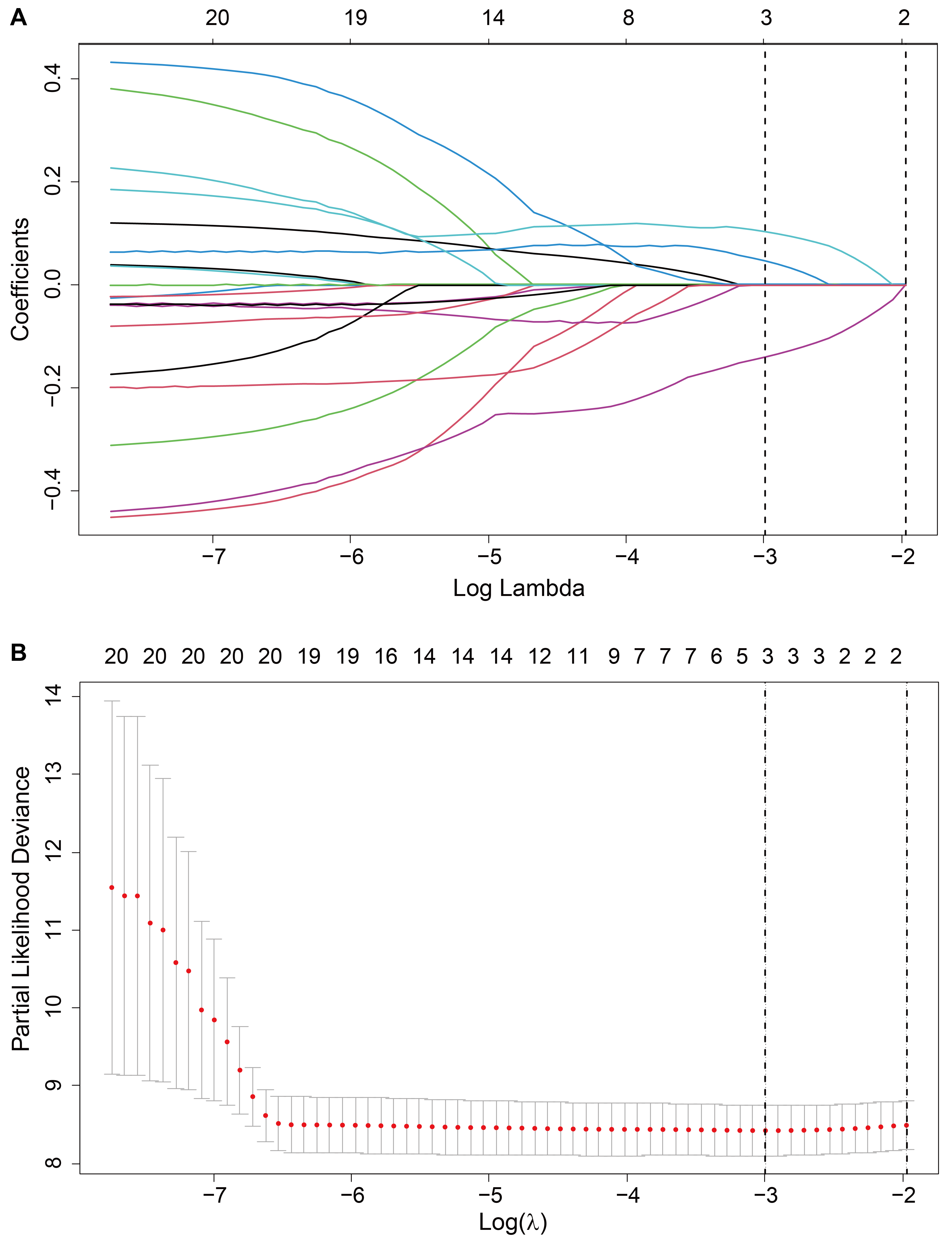

Supplement: Supplemental Information 2 — (A) Coefficient profiles of 20 candidate prognostic variables plotted against log(λ). Each colored line represents the trajectory of a variable’s coefficient as the regularization parameter changes. (B) Ten-fold cross-validation curve showing partial likelihood deviance versus log(λ). The left dashed line indicates the minimum deviance λ, and the right dashed line indicates the largest λ within 1-SE of the minimum, resulting in three non-zero coefficients (PLR, SIRI, PNI) retained for model construction. [file peerj-14-21122-s002.png]
